# Valorisation of Winery By-Products: Revealing the Polyphenolic Profile of Grape Stems and Their Inhibitory Effects on Skin Aging-Enzymes for Cosmetic and Pharmaceutical Applications

**DOI:** 10.3390/molecules29225437

**Published:** 2024-11-18

**Authors:** Rui Dias-Costa, Concepción Medrano-Padial, Raquel Fernandes, Raúl Domínguez-Perles, Irene Gouvinhas, Ana Novo Barros

**Affiliations:** 1 Centre for the Research and Technology of Agro-Environmental and Biological Sciences (CITAB), Institute for Innovation, Capacity Building and Sustainability of Agri-Food Production (Inov4Agro), University of Trás-os-Montes e Alto Douro, Quinta de Prados, 5000-801 Vila Real, Portugal; rgfernandes@utad.pt (R.F.); igouvinhas@utad.pt (I.G.); 2 Laboratorio de Fitoquímica y Alimentos Saludables (LabFAS), Centro de Edafología y Biología Aplicada del Segura-Spanish Council for Scientific Research (EBAS-CSIC), University Campus of Espinardo 25, 30100 Murcia, Spain; cmedrano@cebas.csic.es (C.M.-P.); rdperles@cebas.csic.es (R.D.-P.)

**Keywords:** grape stems, polyphenolic profile, antioxidant capacity, anti-aging, valorisation

## Abstract

Grape (*Vitis vinifera* L.) stems, a by-product of winemaking, possess significant potential value due to their rich polyphenolic composition, which allows their exploitation for cosmetic and pharmaceutical applications. This presents a promising opportunity for valorisation aimed at developing innovative products with potential health-promoting effects. In this study, the polyphenolic profile of extracts from grape stems of seven white grape varieties was determined using spectrophotometric and chromatographic methods, specifically high-performance liquid chromatography coupled with a photodiode array detector and electrospray ionization multi-stage mass spectrometry (HPLC-PDA-ESI-MSn), as well as on their ferric-reducing antioxidant power (FRAP) and radical scavenging capacity, using 2,2-diphenyl-1-picrylhydrazyl (DPPH^●^) and 2,2′-azino-bis(3-ethylbenzothiazoline-6-sulfonic acid (ABTS^●+^) radicals. This study also evaluated the anti-aging activity and skin depigmenting activity of these extracts. These findings revealed a diverse polyphenolic profile, encompassing proanthocyanidins and catechin derivatives (PCDs), phenolic acids, and flavonols. Among the varieties studied, ‘Códega do Larinho’ exhibited the highest concentrations of six distinct polyphenols and the highest total phenolic content. It also demonstrated the highest results for antioxidant capacity and elastase and tyrosinase inhibition. Pearson’s correlation analysis showed a significant positive correlation between certain PCDs with both FRAP and DPPH assays, as well as between the identified flavonols and anti-elastase activity. These results underscore the potential health benefits of grape stem extracts and emphasize the importance of their polyphenolic composition in enhancing antioxidant and anti-aging properties, thus supporting their application in different industries.

## 1. Introduction

In the last year, the production of by-products by the oenological sector has increased, achieving a waste output of around 20 million tons (Mtons) [1,2]. Disposing of materials containing biodegradable organic matter characterized by their acidic pH, salinity, and heavy metal content leads to significant environmental and economic issues, thus impacting the sustainability and competitiveness of the sectorial industries [3,4]. In this context, both the European Green Deal and the International Organisation of Vine and Wine (OIV) (strategic plan 2020–2024) promote circular economy principles by advocating for the reuse of waste and management of by-products through recycling and valorising alternatives that lower these constraints while allowing obtaining new marketable co-products [5]. In this sense, winery by-products (WBPs) harbour significant economic potential as natural sources of bioactive compounds with noteworthy biological benefits that contribute to human health and well-being [6]. Plant extracts obtained from winemaking by-products (grape stems, grape pomace, wine lees, and wine pruning woods (grapevine shoots and grape canes)) [4,7,8,9] have gained recognition across various sectors, namely food (nutraceuticals, functional foods, and additives, among others), pharmaceuticals, and cosmetics [1,7,10,11,12].

Grape (*Vitis vinifera* L.) stems, a lignocellulosic residue that accounts for approximately 5–7% (*w/w*) of the total material processed [4,13], are removed upon destemming operations before vinification to prevent excessive astringency provided by proanthocyanins [2,14]. This by-product is currently processed by landfilling, landfarming, composting, or as a foodstuff and feedstuff [15].

In addition to being a matrix rich in cellulose, hemicellulose, and lignin, the stem has a composition rich in polyphenolic compounds, namely hydroxycinnamic and hydroxybenzoic acids, anthocyanins, flavan-3-*ols*, flavones, flavonols, flavanonols, stilbenes, and proanthocyanidins. However, the polyphenolic profile is influenced by a range of factors, namely the grape variety and the edaphoclimatic conditions [16,17]. These phytochemicals extracted from grape stems are described as being responsible for several biological activities; namely, antioxidant capacity, anti-aging, anti-inflammatory, antimicrobial, anticancer, and antidiabetic activities [1,18,19]. 

Skin aging is a complex, multifactorial process influenced by age, hormonal shifts, genetics, UV exposure, oxidative stress, and inflammation. This process impacts both the epidermal and dermal layers, leading to visible signs such as wrinkles, loss of elasticity, and changes in pigmentation. A critical factor contributing to these changes is the degradation of the extracellular matrix (ECM), a process that is accelerated by enzymes such as matrix metalloproteinases (MMPs), including gelatinases and collagenases and elastolytic enzymes (elastases). These enzymes break down vital components like collagen and elastin, leading to a weakened skin structure and ultimately resulting in the formation of wrinkles and sagging [20,21,22]. Inhibiting these enzymes can slow the visible effects of skin aging, offering a promising strategy for maintaining skin health and appearance and treating skin diseases [21,23,24]. 

There is a growing interest in polyphenols for preventing age-related disorders, highlighting the need for new drug-like molecules, such as natural antioxidants, that can address these conditions. Simultaneously, there is an increasing demand for cosmetic products employing these natural sources to mitigate the effects of skin aging [25,26]. Grape stems, a WBP generated in large quantities in Portugal, could be utilized to develop this type of product.

In this sense, the substantial generation of WBPs in high-producing regions in Portugal highlights the urgent need for robust recovery approaches. Importantly, although various biological activities have been identified in grape stems, their anti-hyaluronidase potential—a relevant factor in anti-aging applications—has not yet been explored, representing a novel aspect of this study. Thus, this research aims to address these WBPs’ valorisation potential, advancing the recovery of these by-products into high-value applications. For that, the present study will explore the comprehensive polyphenolic composition of grape stems from seven Portuguese white grape varieties using advanced HPLC-PDA-ESI-MSn. Beyond identifying their phytochemical profiles, this study will evaluate the functional attributes of their polyphenolic extracts, including their antiradical activity (DPPH^●^ and ABTS^●+^), FRAP, anti-aging activities (anti-elastase and anti-hyaluronidase assays), and skin depigmenting activity (anti-tyrosinase activity). 

## 2. Results and Discussion

### 2.1. Total Phenolic, Ortho-Diphenol, and Flavonoid Content

The total phenolic content (TPC), *ortho*-diphenol content (ODC), and flavonoid content (FC) of grape stems from the white varieties under study were determined, revealing a consistent trend (Table 1), particularly for the ‘Códega do Larinho’ variety, with no significant differences in ODC compared to ‘Síria’ and ‘Gouveio Real’. The TPC ranged from 26.09 to 49.98 mg of gallic acid equivalents per gram of dry weight (mg GAE/g dw), the ODC varied from 25.92 to 40.85 mg GAE/g dw), and the FC ranged from 17.54 to 35.91 mg of catechin equivalents per gram of dry weight (mg CE/g dw). 

On the opposite side, the ‘Moscatel’ variety showed the lowest values, and its *ortho*-diphenol content was not significantly different from that of ‘Viosinho’. These results highlight the variability in phenolic content between varieties. Specifically, the ‘Códega do Larinho’ variety demonstrated the highest levels of TPC and ODC, suggesting that this variety might possess a distinct genetic or physiological characteristic that contributes to its higher phenolic concentrations. In fact, previous studies have shown that grapevine varieties differ in their genetic capacity to produce these compounds, with certain varieties exhibiting higher levels due to specific enzyme activities involved in their biosynthesis. Moreover, physiological characteristics are related with the plant’s overall metabolic activity and its capacity to produce secondary metabolites, influencing their content. These intrinsic factors, when coupled with environmental variables such as climate, soil type, and viticultural practices, likely explain the observed variability in phenolic content across varieties [27,28].

Leal et al. [29] also analysed the TPC of white grape stem varieties, including ‘Rabigato’, ‘Malvasia Fina’, ‘Fernão Pires’, ‘Viosinho’, and ‘Moscatel’. They reported higher TPC values compared to those obtained in the present study (94.71, 123.09, 110.15, 96.99, and 108.71 mg GAE/g dw, respectively). However, for ‘Arinto’ (30.91 ± 0.73 mg GAE/g dw), they found a lower TPC value (Table 1). 

Regarding the ODC, Leal et al. [29] reported higher concentrations of *ortho*-diphenols in grape stems of the white varieties ‘Malvasia Fina’, ‘Viosinho’, and ’Moscatel’ (116.18, 80.62, and 99.38 mg GAE/g dw, respectively) compared to the current study, employing identical extraction conditions. On the other hand, the result for the ‘Arinto’ variety (Table 1) closely matches those reported by Leal et al. [12], at a value of 32.17 mg GAE/g dw. Nevertheless, the ‘Fernão Pires’ variety analysed by the same authors yielded a higher result (69.73 mg GAE/g dw) compared to all other varieties of the present work. 

Concerning the FC, the previous trend was not observed, with the ‘Síria’ variety presenting the highest content, which was significantly different from the others. Moreover, the ‘Viosinho’ variety exhibited the lowest FC (Table 1). In the study performed by Leal et al. [29], the FCs of the ‘Malvasia Fina’, ‘Viosinho’, and ‘Moscatel’ varieties exceeded those found in the present study. Additionally, their analysis revealed higher values for other white varieties, namely ‘Rabigato’ and ‘Fernão Pires’, at 86.22 and 51.37 mg of catechin equivalent per gram of dry weight (mg CE/g dw), respectively. The ‘Moscatel’ varieties analysed by Gouvinhas et al. [1] exhibited higher results for FC (ranging from 34.75 to 73.08 mg CE/g dw) compared to the results of the present work (Table 1). Conversely, the ‘Arinto’ variety displayed a higher content (26.93 ± 1.79 mg CE/g dw) compared to the ‘Arinto’ variety analysed by Leal et al. [12] (25.76 ± 1.14 mg CE/g). Nevertheless, ‘Fernão Pires’ extracts analysed by the same authors exhibited a higher content compared to the white varieties in this study. 

The differences observed in the phenolic content of stem extracts obtained using the same extraction methodologies can be attributed to the distinct intrinsic characteristics of each variety (both genetic and physiological levels), differing stages of ripeness, as well as variations in edaphoclimatic conditions such as climate, soil composition, viticultural practices [12,29,30,31,32,33], and pathogen infections [34]. Ramakrishna et al. [35] reported that several environmental factors, including temperature (heat and cold), salinity, water availability (drought and flooding), radiation (light, UV, and ionizing radiation), chemical stress (mineral salts, gaseous toxins, pollutants, heavy metals, pesticides, and aerosols), and mechanical stress (wind, soil movement, and submersion) can influence the production of secondary metabolites. 

### 2.2. Polyphenolic Profile and Quantification of White Grape Stems

The polyphenolic profile of grape stems from white varieties was tentatively set up using HPLC-PAD-ESI-MS/Mn (Table 2). The analysis of the distribution of individual phenolics in the separate varieties under consideration showed a robust expression of all compounds identified in the seven varieties.

When profiling the individual phenolics, the presence of compounds belonging to the sub-classes of proanthocyanidins and catechin derivatives, phenolic acids, and flavonols was found.

The analysis of the proanthocyanidins and catechin derivatives profile of grape stems provided evidence of the presence of five compounds in concentrations higher than the limit of detection (LOD) of the technique, namely proanthocyanidin dimer digallate (B-type), proanthocyanidin trimer (B-type), catechin-gallocatechin, proanthocyandin dimer (B-type), and catechin (Table 2).

The spectrometry analyses allowed identifying three dimeric proanthocyanidins with [M − H]^−^ pseudo molecular ions at *m/z* 881, 593, and 577 amu, corresponding to proanthocyanidin dimer digallate (B-type), catechin-gallocatechin, and proanthocyandin dimer (B-type), respectively, according to the previous descriptions by Costa et al. [17], and Matos et al. [22]. After fragmentation, proanthocyanidin dimer digallate (B-type) displayed MS2 spectra including the product ions *m/z* 695 amu corresponding to the loss of a water molecule from the Diels Alder fission in the C ring (*m/z* 713 amu). Catechin-gallocatechin displayed MS2 spectra including product ions with m/z 441 amu [M – H − 152]^−^ (galloyl moiety) and 423 amu [M − H − 152 − 18]^−^ (galloyl moiety and water molecule) [23]. Finally, the proanthocyanidins dimers included the proanthocyanidin dimer (B-type) that beyond the pseudo molecular ion at *m/z* 577 amu, the MS2 spectra included product ions with *m/z* 425 amu [M − H − 152]^−^ (galloyl moiety) and 407 amu [M − H − 152 − 18]^−^ (galloyl moiety and water molecule), as well as *m/z* 289 amu, corresponding to the quinone methide or interflavan bond cleavage. In addition, the fragmentation of proanthocyanidin trimer (B-type) gave rise to fragment ions at *m*/*z* 695 amu [M − H − 170]^−^ and *m/z* 577 amu [M − H − 288]^−^, which corresponds to the loss of gallic acid and identify the trimmer upper subunit, correspondingly.

Concerning phenolic acids, the grape stems of white varieties displayed two compounds in concentrations higher than the limit of detection (LOD) of the technique namely *trans*-caftaric acid and *trans*-coutaric acid (Table 3), which were identified resorting to the [M − H]^−^ pseudo molecular ions (at *m/z* 311 and 295 amu, respectively) and the fragment ions at *m/z* 149, 179, and 135, corresponding to fragments of tartaric acid, caffeic acid, and a low signal produced by the caffeic acid decarboxylation, respectively, and at *m/z* 163 amu for *trans*-coutaric acid. Matos et al. [22] also obtained the fragment ion at *m/z* 163 of *trans*-coutaric acid and the fragment ions at *m/z* 179, 149, 135 of caftaric acid in grape pomace and wine lees. Similarly, Costa-Pérez et al. [17] detected the same fragment ions for caftaric acid. 

The individual flavonols identified were quercetin-3-*O*-glucuronide and kaempferol-3-*O*-glucoside, according to their [M − H] pseudo molecular ions (*m/z* at 477 and 447 amu, respectively). Thus, the former fragmented to give rise to an *m/z* 301 amu corresponding to unesterified quercetin by losing the glucuronide moiety ([M − H − 176]^−^), while kaempferol glucoside fragmented to provide a base peak at *m/z* 285 amu ([M − H − 162]^−^, corresponding to the loss of a glucose moiety) (Table 2). The fragments ions of the quercetin-3-*O*-glucuronide are in accordance with the findings of Costa-Pérez et al. [17], Matos et al. [22], Peixoto et al. [36], and Delgado de la Torre et al. [37].

Regarding proanthocyanidins and catechin derivatives, although nine individual compounds were detected above the LOD and thus could be properly identified, only eight compounds were present at concentrations exceeding the limit of quantification (LOQ). Table 3 presents the quantification of polyphenolic compounds in the seven white stems, analysed by HPLC-DAD-MS/Mn.

The proanthocyanidins and catechin derivatives were found in the following decreasing order of concentration (total concentration per variety): ‘Moscatel’ (3163.02 mg/g dw) > ‘Arinto’ (2934.67 mg/g dw) > ‘Síria’ (2866.00 mg/g dw) > ‘Códega do Larinho’ (2850.91 mg/g dw) > ‘Gouveio Real’ (2621.28 mg/g dw) > ‘Malvasia Fina’ (2214.90 mg/g dw) > ‘Viosinho’ (2022.90 mg/g dw) (Table 3). The concentration of each identified compound of the seven grape stem varieties of this study was revealed to be significantly higher than the ones quantified by Costa et al. [17], which averaged concentrations recorded in all matrices (stems, pomace, and wine lees) ranging between 0.01 and 0.10 mg/g dw. 

The proanthocyanidin dimers (B-type) Isomer 1 and Isomer 2 and the proanthocyanidin trimers (B-type) Isomer 1 and Isomer 2 studied by Milinčić et al. [16] showed lower results (52.90, 1.60, 2.20, and 5.40 µg/g dw, respectively) compared to the compounds quantified in the present work (Table 3). Milinčić et al. [16] also quantified catechin in stems at a concentration lower than the ones measured in the present study, measuring 25.90 µg/g dw. On the other hand, the catechin concentration in the samples (487.97 µg/g dw, on average) is lower than the average concentration found in grape stem samples from the works performed by Jara-Palacios et al. [38], Anastasiadi et al. [39], and González-Centeno et al. [30] (688.00, 872.00, 654.40 µg/g dw, respectively).

In regard to phenolic acids, namely *trans*-caftaric acid and *trans*-coutaric acid, the concentration decreased as follows ‘Moscatel’ (310.19 mg/g dw) > ‘Códega do Larinho’ (227.62 mg/g dw) > ‘Viosinho’ (197.02 mg/g dw) > ‘Arinto’ (145.69 mg/g dw) > ‘Gouveio Real’ (90.57 mg/g dw) > ‘Síria’ (89.02 mg/g dw) > ‘Malvasia Fina’ (75.82 mg/g dw). Jara-Palacios et al. [38] and Anastasiadi et al. [39] also quantified *trans*-caftaric acid in stems, reporting lower values (168.00 and 723.00 µg/g dw, on average, respectively) compared to the present findings (Table 3). Regarding *trans*-coutaric acid, Jara-Palacios et al. [38] obtained a lower concentration (20.70 µg/g dw) compared to the samples in this study.

Regarding flavonols, specifically quercetin-3-*O*-glucuronide, there were no significant differences among the varieties, with ‘Síria’ exhibiting the highest concentration. Peixoto et al. [36] studied other winery by-products, such as skins, seeds, and their mixture, and found lower concentrations of quercetin-3-*O*-glucuronide compared to the stem samples in this study (343.50, 253.00, and 889.00 µg/g dw, respectively).

In relation to kaempferol-3-*O*-glucoside, only ‘Códega do Larinho’ was significantly different from the other varieties, showing the lowest concentration. Milinčić et al. [16] presented an average concentration of this compound in grape stem samples lower (9.30 µg/g dw) than all the analysed varieties (Table 3).

As expected, and consistent with the results from spectrophotometric methods, there are differences between these findings and those of other studies. Once again, these differences in the concentration of polyphenolic compounds may be due to grape variety, cultivation conditions, stage of ripeness, harvesting and processing methods, storage and preservation, extraction and analysis methods, and biochemical interactions.

### 2.3. Radical Scavenging Capacity and Reducing Power of Grape Stems’ Polyphenols

The analysis of the antioxidant capacity of the polyphenolic extracts of grape stems provides very useful information to establish the actual healthy properties of this plant material. Thus, the assessment of grape stems’ polyphenols and their radical scavenging and reducing capacities provides robust evidence concerning their functional scope and allows the performance of comparative analyses.

According to DPPH^●^ and ABTS^●+^ scavenging outcomes and reducing power (FRAP), it was observed that grape stems from ‘Códega do Larinho’ presented the highest values using the three methodologies (0.32 ± 0.02, 0.46 ± 0.03, and 0.34 ± 0.01 mmol of Trolox equivalent per gram mmol TE/g dw, respectively) (Table 4). 

Using the DPPH methodology, the ‘Códega do Larinho’ variety was significantly different from all the other varieties. In the ABTS and FRAP methodologies, it was significantly different from all others, except for ‘Síria’. Conversely, the ‘Viosinho’ variety exhibited the lowest values for DPPH^●^ radical scavenging and reducing power, at 0.12 ± 0.01 and 0.21 ± 0.00 mmol TE/g dw, respectively, standing out as significantly different from the other samples. Regarding the ABTS methodology, ‘Arinto’ showed the lowest values, which were not significantly different from those of the ‘Malvasia Fina’ and ‘Gouveio Real’ varieties.

The free radical scavenging activity values for both DPPH^•^ and ABTS^•+^ radicals were lower than those reported for the ‘Malvasia Fina’ and ‘Viosinho’ varieties in a study by Leal et al. [29] (Table 4). On the other hand, grape stem extracts from the ‘Moscatel’ variety showed lower values in comparison to those reported by Leal et al. [29], who reported 0.42 mmol TE/g dw for DPPH and 1.17 mmol TE/g dw for ABTS. The ‘Arinto’ variety analysed by the same authors exhibited a notably higher ABTS^•+^ scavenging capacity (0.35 ± 0.00 mmol TE/g dw) compared to the results of the present work. In the same study, the ‘Rabigato’ and ‘Fernão Pires’ varieties exhibited higher free radical scavenging activity, as determined by the ABTS and DPPH methods, compared to the samples analysed in this study. Contrary to what was observed previously, the results for the ‘Moscatel’ and ‘Malvasia Fina’ varieties in the DPPH and ABTS assays showed higher values than those reported by Perles et al. [40] (‘Malvasia Fina’: 0.01 mmol TE/g dw for DPPH and 0.02 mmol TE/g dw for ABTS; ‘Moscatel’: 0.02 mmol TE/g dw for DPPH and 0.02 mmol TE/g dw for ABTS). The same happens when comparing the DPPH results for the ‘Arinto’ variety (Table 4), which are higher than those reported by Leal et al. [29] (0.15 ± 0.01 mmol TE/g dw). 

In light of the comparison between these findings and the literature applying identical extraction methodologies and antioxidant assays, it can be deduced that the antioxidant capacity of the ‘Arinto’ variety, as measured by the FRAP assay (Table 4), is lower than that reported by Leal et al. [29] (0.35 ± 0.02 (mmol TE/g dw), whereas the Fernão Pires variety (0.99 ± 0.02 mmol t/g dw), analysed by the same authors, demonstrated a higher value than all other white varieties. Based on our knowledge, this study is the first to analyse the radical scavenging capacity and reducing power using the three methodologies applied in this research for the ‘Códega do Larinho’, ‘Síria’, and ‘Gouveio Real’ varieties. 

Once again, the variations in the antioxidant capacity of stem extracts can be attributed to genetic and physiological differences, stages of ripeness, edaphoclimatic conditions, and environmental factors such as temperature, salinity, water availability, radiation, chemical stress, and mechanical stress. The choice and combination of solvents, along with extraction parameters like temperature, time, pH, solid-to-liquid ratio, and pressure, also significantly influence the antioxidant capacity of this material.

### 2.4. Anti-Aging Activity (Anti-Elastase and Anti-Hyaluronidase Activities)

The search for natural alternatives with skin anti-aging properties has significantly increased, with the focus turned on bioactive compounds derived from agro-industrial by-products. The exploration of these by-products, rich in polyphenolic compounds, opens new opportunities for the development of cosmetic products with functional properties with potential skin benefits [18,22,41,42,43]. 

**Figure 1:**
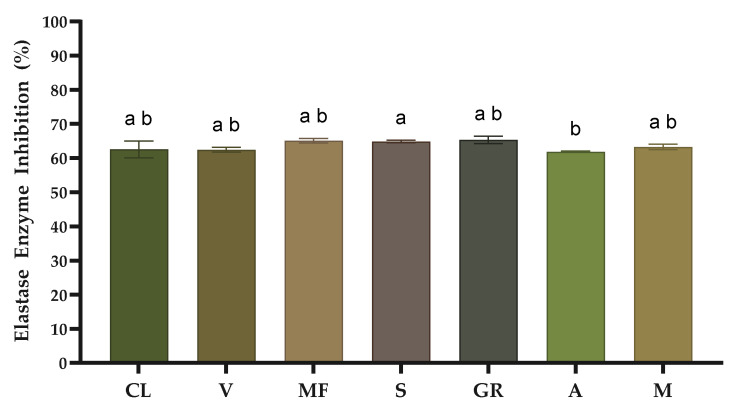
Anti-elastase activity of grape stem extracts at 1 mg/mL. CL, ‘Códega do Larinho’; V, ‘Viosinho’; MF, ‘Malvasia Fina’; S, ‘Síria’; GR, ‘Gouveio Real’; A, ‘Arinto’; M, ‘Moscatel’. Data are presented as mean ± SD (*n* = 3). Different letters correspond to significant differences between varieties (*p* < 0.05) according to a Welch test followed by a Dunn post hoc test.

**Figure 2:**
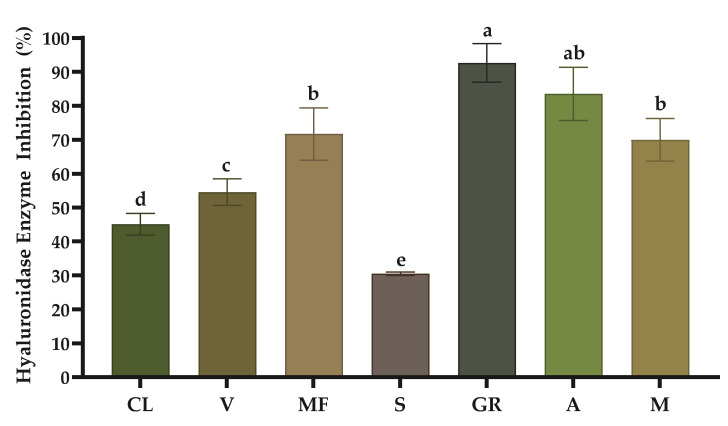
Anti-hyaluronidase activity of grape stem extracts at 1 mg/mL. CL, ‘Códega do Larinho’; V, ‘Viosinho’; MF, ‘Malvasia Fina’; S, ‘Síria’; GR, ‘Gouveio Real’; A, ‘Arinto’; M, ‘Moscatel’. Data are presented as mean ± SD (*n* = 3). Different letters correspond to significant differences between varieties (*p* < 0.05) according to an ANOVA test followed by Tukey’s post hoc test.

The enzymes elastase and hyaluronidase contribute to skin aging, causing the degradation of elastin fibres and hyaluronic acid, respectively. The inhibition of these enzymes is considered an efficient strategy to combat skin aging, such as loss of elasticity, skin hydration and occurrence of wrinkles. In the present study, we assessed the inhibitory activity of the seven grape stem extracts on the referred enzymes to investigate their anti-aging potential. Figure 1 shows the results of elastase enzyme inhibition, and Figure 2 displays the results of hyaluronidase enzyme inhibition.

The elastase enzyme inhibition results demonstrated a promising activity of grape stem extracts at 1 mg/mL concentration, where the inhibition values ranged from 61.84% to 65.35%. There are no significant differences between the white grape stem varieties. The results of this study are lower than those presented by Leal et al. [18], who reported inhibition percentages of 72.93% and 67.98% for the white varieties ‘Fernão Pires’ and ‘Arinto’, respectively. Comparing these inhibition percentages with other WBPs, it was possible to conclude that the results for the seven white grape stems are slightly lower compared with those presented by Wittenauer et al. [41], who analysed grape pomace. 

In terms of hyaluronidase enzyme inhibition, the grape stem extracts also displayed a notable enzyme inhibition, namely the ‘Gouveio Real’ variety, which is significantly different from other stems, except the ‘Arinto’ variety, making it a promising candidate for use in the cosmetic industry to combat skin aging. To the best of our knowledge, there are no studies in the literature that analyse hyaluronidase enzyme inhibition using WBP extracts. 

Several studies have reported that the extracted polyphenolic compounds from winery by-products exhibit several cosmetics interests. In the case of grape pomace, the isolated compounds resveratrol, catechin, and gallic acid were described as having anti-aging functions. Regarding the grape stems and grape seed, epicatechin was described as having anti-aging functions [44,45,46]. The polyphenolic compounds extracted from the grape varieties of the present study were shown to have anti-aging effects. Among the compounds, catechin helps maintain the skin’s extracellular matrix by promoting the breakdown of old or damaged collagen through the increase of collagenase matrix metalloproteinase-1 (MMP-1) while simultaneously slowing down the production of new collagen to balance the overall process [47]. Biondi et al. [48] proved that proanthocyanidins extracted from grape seeds inhibited MMP-1. Liu et al. [49] demonstrated that *Sea buckthorn* proanthocyanidins can promote the synthesis of type I collagen in aged human skin fibroblasts and inhibit the degradation of type I collagenase by regulating the MMPs inhibitory system, thereby maintaining the stability of the extracellular matrix structure to achieve anti-aging purposes. In the work performed by Zagórska-Dziok et al. [50], caftaric acid and quercetin-3-*O*-glucuronide inhibited the activity of elastase. Although resveratrol has not been identified in the samples used in this study, it is known for its ability to penetrate the skin barrier, stimulate fibroblast proliferation, and enhance collagen III concentration.

These documented beneficial effects led us to evaluate the skin anti-aging potential of the extracted compounds through primary in vitro assays.

### 2.5. Skin Depigmenting Activity (Anti-Tyrosinase Activity)

Similar to the growing interest in natural compounds with anti-aging properties, the quest for alternatives in skin depigmentation is also notable.

Tyrosinase is an enzyme that catalyses the conversion of tyrosine to melanin, playing a crucial role in the rate-limiting step that regulates melanin production. However, the overproduction of melanin in the skin may cause hyperpigmentation and melanoma and could be genotoxic. Therefore, the inhibition of tyrosinase activity tends to induce skin whitening due to a reduction of melanin synthesis [21,51]. Catechins, quantified in the present study, have been reported to exhibit depigmenting effects. These effects are associated with the direct inhibition of tyrosinase activity and the downregulation of its expression in B16 melanoma cells [24,52]. Concerning proanthocyanidins, a study by Chai et al. [53] revealed that these compounds can strongly inhibit the monophenolase and diphenolase activities of tyrosinase. Honisch et al. [54] concluded that *trans*-caftaric acid, one of the phenolic acids extracted in this study, serves as a competitive inhibitor of tyrosinase and is more potent than the related caffeic and chlorogenic acids. This finding highlights its potential applications in the cosmetic and food industries, particularly for developing natural skin whitening formulations and to counteract enzymatic browning in food products.

The remaining compounds detected in this study, particularly flavonols, have been reported to inhibit tyrosinase activity. Karim et al. [55] reported that quercetin is more effective at inhibiting the tyrosinase enzyme compared to kaempferol. In a study conducted by Quispe et al. [56], quercetin exhibited significant inhibition of tyrosinase, surpassing the effects of kojic acid, which was used as the control. These compounds have been shown to interfere with activity by chelating copper at the enzyme’s active site, thereby suppressing melanogenesis either through the direct inhibition of tyrosinase activity or by reducing its expression [57,58].

Other phytochemicals not extracted in this study, such as arbutin, resveratrol, ellagic acid, and gentisic acid, have been shown to inhibit several steps in the melanogenic pathway. These compounds are recognized for their ability to support skin stability, provide anti-aging benefits, preserve skin health, and act as moisturizers [59].

**Figure 3:**
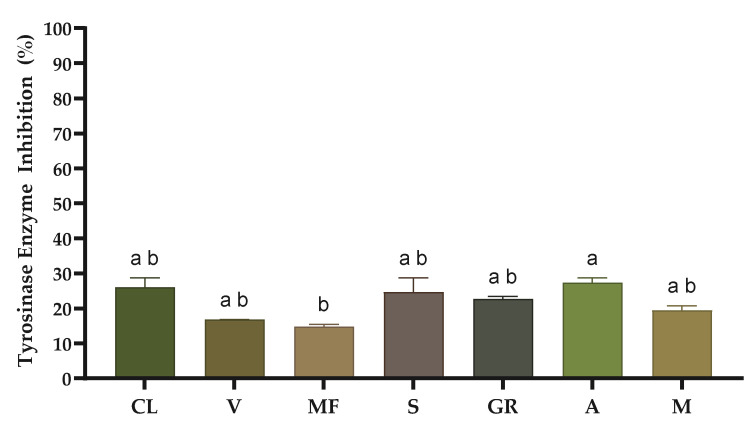
Anti-tyrosinase activity of grape stem extracts at 1 mg/mL concentration. CL, ‘Códega do Larinho’; V, ‘Viosinho’; MF, ‘Malvasia Fina’; S, ‘Síria’; GR, ‘Gouveio Real’; A, ‘Arinto’; M, ‘Moscatel’. Data are presented as median with range (*n* = 3). Different letters correspond to significant differences between varieties (*p* < 0.05) according to a Kruskal–Wallis test followed by a Dunn post hoc test.

The results of the enzymatic inhibition of tyrosinase are shown in Figure 3, ranging from 14.76% in ‘Malvasia Fina’ to 27.75% in ‘Arinto’. Notably, only the ‘Arinto’ and ‘Malvasia Fina’ varieties exhibit significantly different anti-tyrosinase activity. The results of the current work are lower compared to the study by Leal et al. [18] which analysed two white grape stems (‘Arinto’: 44.07 and ‘Fernão Pires’: 44.83%). 

### 2.6. Principal Component Analysis 

#### Polyphenolic Content and Antioxidant Capacity

Principal Component Analysis (PCA) constitutes an essential mathematical approach for visualization and dimensionality reduction that transforms a high-dimensional pool of data into an alternative of lower dimensions. Nonetheless, interestingly, this conversion preserves as much information as possible, resulting in new pools of data that allow for understanding the relationship between the polyphenolic content and the radical scavenging and reducing capacities of a given extract (e.g., the polyphenolic extracts of grape stems).

**Figure 4:**
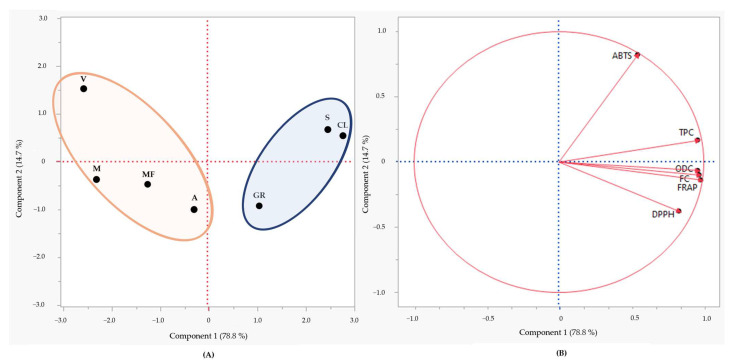
Principal Component Analysis (PCA) scores (**A**) and loadings (**B**) plots of the polyphenolic content (TPC, total phenolic content; ODC, *ortho*-diphenol content, and FC, flavonoid content) and antioxidant capacity (FRAP, ferric-reducing antioxidant power; DPPH, DPPH^•^ scavenging capacity; ABTS, ABTS^•+^ scavenging capacity) of grape stems obtained of the seven white varieties assessed (CL, ‘Códega do Larinho’; V, ‘Viosinho’; MF, ‘Malvasia Fina’; S, ‘Síria’; GR, ‘Gouveio Real’; A, ‘Arinto’; M, ‘Moscatel’).

According to the PCA results presented in Figure 4, the PC1 and PC2 components accounted for 78.8% and 14.7% of the loading score, respectively. Within both the upper and lower right quadrants, a distinct grouping emerges, comprising the grape stems of the varieties ‘Síria’, ‘Códega do Larinho’, and ‘Gouveio Real’.

This distribution is in good agreement with the high values recorded for almost all parameters monitored (total phenolics, *ortho*-diphenols, and flavonoids, DPPH^•^ and ABTS^•+^ scavenging capacity, and FRAP-based reducing power) concerning grape stems. In contrast, the remaining varieties exhibited the opposite distribution, being located into both upper and lower left quadrants pulled by the lower values exhibited concerning the lower values recorded for the parameters monitored.

### 2.7. Pearson Correlations Between the Polyphenolic Composition, Antioxidant Capacity, and Anti-Aging and Skin Depigmenting Activities

**Figure 5:**
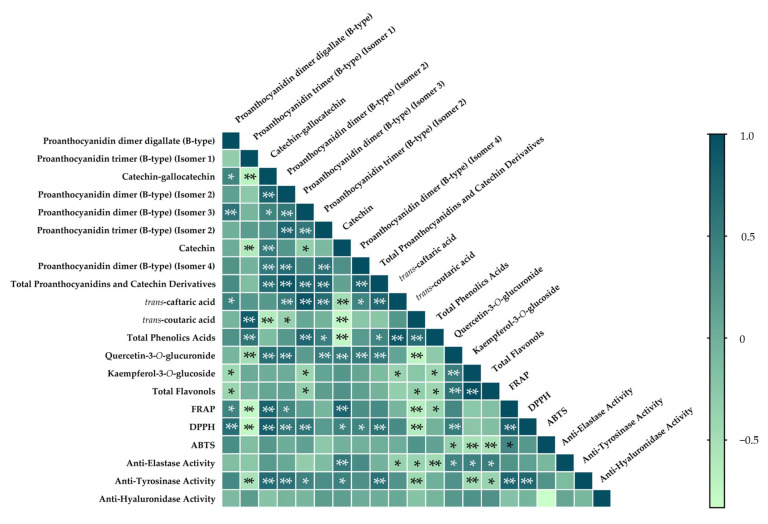
Heatmap of correlations (r) between the polyphenolic composition, radical scavenging capacity (DPPH^•^ and ABTS^•+^) reducing power antioxidant capacity using FRAP assay, and anti-elastase, anti-tyrosinase, and anti-hyaluronidase activities. Statistically significant correlations: * *p* < 0.05, ** *p* < 0.01.

When analysing the correlation existing between the phytochemical composition of different plant materials and their scavenging and reducing powers, as well as the inhibition of elastase, hyaluronidase, and tyrosinase using Pearson’s correlation, significant results were obtained (Figure 5).

By the analysis of Figure 5, it is possible to observe that, overall, the compounds with the highest concentration (Table 3), namely the total proanthocyanidins and catechin derivatives, showed significantly positive correlations with FRAP or DPPH assays, with proanthocyanidin dimer digallate (B-type), catechin-gallocatechin, proanthocyanidin dimer (B-type) (Isomer 2), and catechin standing out. It is also important that of the compounds belonging to “total proanthocyanidins and catechin derivatives”, no significant correlations were observed with the FRAP and ABTS methodology. In the class of flavonols, only quercetin-3-*O*-glucoside correlated positively and significantly with antioxidant capacity using DPPH methodology. 

The significant positive correlation between catechin and the FRAP methodology found by Jara-Palacios et al. [8], which examined wine lees, corroborates the correlation observed in this study. In contrast, Apostolou et al. [60] did not observe a significant positive correlation with the DPPH methodology. Regarding the ABTS assay, they also did not observe a correlation, which is similar to the present findings. Melo et al. [7], who analysed pomace and rachis, did not uncover a significant correlation with the DPPH assay, in contrast to the findings in this study (*p* < 0.05).

In terms of the correlations between phenolic acids and antioxidant capacity, Apostolou et al. [60] found a negative correlation between *trans*-caftaric acid and the ABTS assay, a result that was not verified in this study. 

Similar to the present work, some studies [31,40] found significant positive correlations between quercetin-3-*O*-glucuronide and the inhibition of the DPPH radical. 

The correlations obtained in this study between quercetin-3-*O*-glucuronide and DPPH radical inhibition, as well as kaempferol-3-*O*-glucoside and the ABTS assay, were significant (*p* < 0.01). 

In some cases, no correlation was found between specific polyphenols and the antioxidant capacity of the samples. This suggests that antioxidant capacity is related to the total polyphenolic content rather than to individual compounds, even though certain polyphenols may contribute more significantly to the overall antioxidant capacity in each type of sample.

Significant positive correlations were also found between anti-elastase activity and several compounds; namely, catechin, quercetin-3-*O*-glucuronide, kaempferol-3-*O*-glucoside, and total flavonols. Several studies have also explored the interactions between elastase and catechin as inhibitors [51]. According to Anggraini et al. [61] and Malarenko et al. [62], quercetin can deactivate protease activity and is the most effective inhibitor of elastase release. Jakimiuk et al. [63] also reported that this flavonol has been used in several studies as a reference compound with a demonstrated inhibitory effect on elastase. The compounds that are constituted by a catechol group exhibit significant inhibitory activity, which is reduced when methylation of one of these groups occurs. On the contrary, compounds without catechol groups have a weak inhibitory effect on elastase action. The inhibitory effect of this enzyme may also be related to the presence of a double bond between carbons C-2 and C-3 in the C ring of flavonoids [63]. These findings support the positive correlation between flavonols and elastase inhibition.

Regarding the correlations between anti-tyrosinase activity and polyphenolic compounds, significant positive correlations exist among catechin-gallocatechin, proanthocyanidin dimer (b-type) Isomers 2 and 3, and total proanthocyanidins and catechin derivatives. In contrast, Xiong et al. [64] concluded that individual catechins, when isolated, did not exhibit anti-tyrosinase activity. However, the results of this study revealed a significant positive correlation. Since proanthocyanidins belong to condensed tannins, the positive and significant correlations obtained between the proanthocyanidins in this study and anti-tyrosinase activity support the findings of Deng et al. [65]. 

Liu et al. [66] reports that other flavonols, such as quercetin-3-*O*-rhamnoside, kaempferol-3-*O*-galactoside, showed lower correlations with tyrosinase inhibitory activities, which aligns with the present findings. Quercetin also exhibited low potential as a tyrosinase inhibitor in the study conducted by Chang et al. [67].

Regarding the correlations between antioxidant capacity and anti-tyrosinase activity, the study by Nguyen et al. [68] supports the results of the current study. 

## 3. Materials and Methods

**Figure 6:**
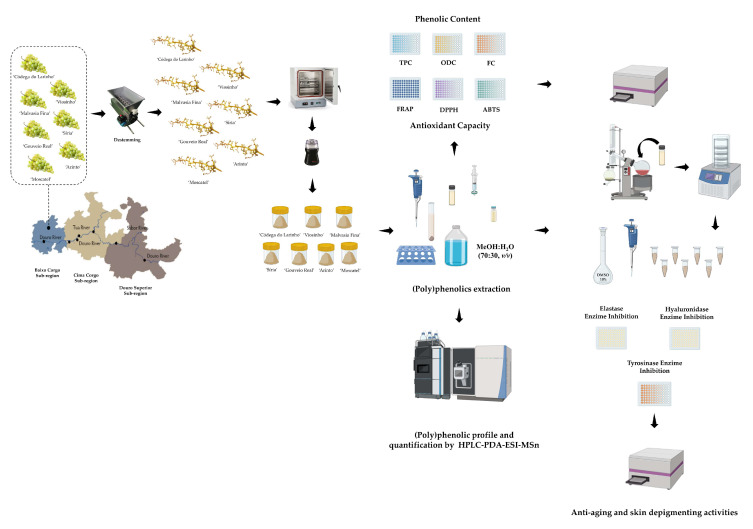
Schematic representation of the methodology used in this study.

The methodology used in this study is represented in Figure 6.

### 3.1. Chemicals and Reagents

Potassium hydroxide (KOH), Folin–Ciocalteu’s reagent, gallic acid (3,4,5-trihydroxybenzoic acid), acetic acid (CH_3_COOH), formic acid (HCOOH), and sodium hydroxide (NaOH) were obtained from Panreac Química SLU (Barcelona, Spain). Hydrochloric acid (HCl), sodium nitrite (NaNO_2_), aluminium chloride (AlCl_3_), sodium carbonate (Na_2_CO_3_), and N-cetylpyridinium chloride, Tris-HCl (C_4_H_12_ClNO_3_) were obtained from Merck (Darmstadt, Germany). Methanol (CH_3_OH) and ABTS^•+^ (2,2-azino-bis(3-ethylbenzothiazoline-6-sulfonic acid) diammonium salt were purchased from VWR (Carnaxide, Portugal). Sodium molybdate (Na_2_MoO_4_) was acquired from Chem-Lab N.V. (Zedelgem, Belgium). Moreover, catechin (C_15_H_14_O_6_), Trolox (6-hydroxy-2,5,7,8-tetramethylchroman-2-carboxylic acid), potassium persulfate (K_2_S_2_O_8_), TPTZ (2,4,6-Tripyridyl-s-Triazine), iron (III) chloride (FeCl_3_), N-Succinyl-Ala-Ala-Ala-p-nitroanilide, potassium phosphate (K_3_PO_4_), dimethyl sulfoxide (DMSO), and the enzymes elastase, hyaluronidase, and tyrosinase, were obtained from Sigma-Aldrich (Steinheim, Germany). DPPH^•^ (2,2-diphenyl-1-picrylhydrazyl radical) was obtained from Alfa Aesar (Porto Salvo, Portugal), while acetonitrile was supplied by J.T. Baker (Philipsburg, NJ, USA). Hyaluronic acid sodium salt from rooster comb was obtained from Frilabo (Maia, Portugal), kogic acid from Acros Organics (Antwerpen, Belgium), and L-tyrosine from Scharlau (Barcelona, Spain). Distilled water from Millipore (Bedford, MA, USA) was used for all extractions and spectrophotometric analysis. The ultrapure water used in the chromatographic analyses was obtained using a Millipore water purification system. 

### 3.2. Sampling

During the 2023 harvest, grape (*Vitis vinifera* L.) stems of seven white varieties, namely ‘Códega do Larinho’, ‘Viosinho’, ‘Malvasia Fina’, ‘Síria’, ‘Gouveio Real’, ‘Arinto’, and ‘Moscatel’, were sourced from the Baixo Corgo sub-region of the *Região Demarcada do Douro* (Table 5). The destemming process was carried out at the winery industry Rozés (Lamego, Portugal). 

### 3.3. Plant Material Processing and Preparation of Polyphenolic Extracts of Grape Stems

The grape stems were dried in an oven (Memmert, Schwabach, Germany) at 40 °C for 72 h, until constant weight. After drying, the plant material was ground into a powder. The extracts were prepared by adding 15 mL of methanol/water (70:30, *v/v*) to 400 mg of sample. Then, the mixtures were thoroughly homogenized for 30 min using an orbital shaker (GFL 3005, GEMINI, Apeldoorn, The Netherlands) and centrifuged at 40,000× *g* for 15 min (Kubota Corporation, Hongo Bunkyo, Tokyo, Japan). This extraction process was repeated three times for each sample and the final volume was adjusted to 50 mL using a volumetric flask (Linex, Marinha Grande, Portugal) with the extraction solvent. For chromatographic and spectrophotometric analyses, the extracts were filtered through 0.2 µm polyvinylidene difluoride syringe filters (Teknokroma, Barcelona, Spain). The filtered extracts were stored at –80 °C until analysis.

### 3.4. Determination of Phenolic Content

The phenolic content of the hydro-methanolic extracts of grape stems was assessed concerning the total phenolics, *ortho*-diphenols, and flavonoids through spectrophotometric techniques, following the protocols outlined by Leal et al. [29] with minor modifications. Absorbance was measured using a spectrophotometer (Thermo Spectronic Genesys 10-S, Rochester, NY, USA) using cuvettes (10 × 35 mm) for the UV-*Vis* spectrophotometric assay (Thermo Fisher Scientific, Shanghai, China). The data were presented as the mean of three replicates (*n* = 3) ± standard deviation. 

#### 3.4.1. Total Phenolic Content

The total phenolic content was determined by mixing 1 mL of the sample/standard with 0.5 mL of Folin–Ciocalteu reagent and adding into the mixture 2 mL of Na_2_CO_3_ (7.5%, *w/v*) and 6.5 mL of water. The mixture was incubated at 70 °C for 30 min, protected from light to prevent photodegradation. Absorbance readings were taken at 750 nanometers (nm), using gallic acid as the standard (concentration range: 5–200 mg/L) (y = 0.0132x + 0.0092; R^2^ = 0.9978). The results were reported as mg of gallic acid equivalents per gram of dry weight (mg GAE/g dw).

#### 3.4.2. *Ortho*-Diphenol Content

The *ortho*-diphenol content was determined by mixing 4 mL of the sample/standard solution with 1 mL of Na_2_MoO_4_ (5%, *w/v*). The mixtures were vortexed and then incubated at room temperature, protected from light, for 15 min. Absorbance was measured at 375 nm and the *ortho*-diphenol content was calculated using a gallic acid standard curve (concentration range: 5–200 mg/L) (y = 0.0119x − 0.0467; R^2^ = 0.9999). The results were expressed as mg of gallic acid equivalents per gram of dry weight (mg GAE/g dw).

#### 3.4.3. Flavonoid Content

The flavonoid content was determined by adding 500 µL of the sample/standard to 150 µL of NaNO_2_ (5.0%, *w/v*). After five minutes, 150 µL of AlCl_3_ (10.0%, *w/v*) was added and the mixture was allowed to react for 6 min. Subsequently, 1 mL of NaOH (1.0 M) was added, followed by shaking for 30 s. The absorbance of the resulting solution was measured at 510 nm. The flavonoid content was calculated using a catechin standard curve (concentration range: 5–200 mg/L) (y = 0.0097x − 0.0549; R^2^ = 0.9995). The results were expressed as mg of catechin equivalents per gram of dry weight (mg CE/g dw).

### 3.5. Radical Scavenging Capacity and Reducing Power

The radical scavenging capacity of the extracts was assessed using two methods: the 2,2-diphenyl-1-picrylhydrazyl (DPPH) assay and the 2,2′-azino-bis(3-ethylbenzothiazoline-6-sulfonic acid (ABTS) assay. Additionally, the reducing activity was measured using the ferric-reducing antioxidant power (FRAP) assay. These methodologies were adapted to a microscale format using 96-well microplates (PrimeSurface MS-9096MZ, Frilabo, Maia, Portugal), according to Leal et al. [29]. Absorbance measurements were conducted using a microplate reader (Multiskan GO Microplate Photometer, Thermo Fisher Scientific, Vantaa, Finland). The data were presented as the mean ± standard deviation.

#### 3.5.1. DPPH Radical Scavenging Assay

Polyphenolic extracts or standard solutions (10 µL) were mixed with 190 µL DPPH working solution and left to react for 30 min, at room temperature, in darkness. After that, the absorbance was measured at 520 nm. Methanol/dH_2_O (70:30, *v/v*) served as a blank. The scavenging capacity of the extracts was determined using Trolox as a reference antioxidant compound (0.039–1.250 mmol/L range of concentrations). The results were expressed as mmol of Trolox equivalents per gram of dry weight (mmol TE/g dw). The percentage of inhibition and Trolox Equivalent Antioxidant Capacity (TEAC) of the samples were calculated as follows:

% inhibition = 100 × (Abs_520_ blank − Abs_520_ sample)/Abs_520_ blank (1)

TEAC (mmol t/g dw) = (% inhibition − b)/a(2)

where a is the slope of the standard curve (y = ax + b) and b is the y-intercept.

#### 3.5.2. ABTS Radical Scavenging Assay

The assessment of the polyphenolic extracts of grape stems on the ABTS^●+^ scavenging capacity was developed by mixing the extracts (12 µL) with 188 µL of the ABTS working solution (prepared by combining 5 mL of ABTS stock solution (7.0 mM in dH_2_O) with 88 µL of potassium persulfate (148 mM) and diluted with sodium acetate buffer (20 mM, pH 4.5). The mixture was allowed to react for 30 min, at room temperature, protected from light. A mixture of 188 µL of the ABTS working solution and 12 µL of dH_2_O served the blanks. Absorbance was measured at 734 nm, and the radical scavenging capacity was expressed as mmol TE/g dw, using a standard curve of Trolox prepared in the range of concentrations 0.034–0.200 mmol/L. The % inhibition and Trolox Equivalent Antioxidant Capacity (TEAC) of the samples were calculated using the following equations:

% inhibition = 100 × (Abs_734_ blank − Abs_734_ sample)/Abs_734_ blank (3)

TEAC (mmol t/g dw) = (% inhibition − b)/a(4)

#### 3.5.3. Ferric-Reducing Antioxidant Power Assay

To determine the antioxidant capacity using this method, 180 µL of the FRAP working solution, including 1 volume of TPTZ (10 mM dissolved in HCl), 1 volume of FeCl_3_ (20 mM in water), and 10 volumes of acetate buffer (8.58 mL of acetic acid in 500 mL of dH_2_O, adjusted the pH 3.6) were mixed with 20 µL of the polyphenolic extracts. The FRAP working solution was prepared daily and warmed at 37 °C for 10 min before use. Then, the mixtures were shaken and incubated at 37 °C in the dark for 30 min. The absorbance was measured at 593 nm using a microplate reader. Concentrations from 0.039 mmol/L to 1.250 mmol/L of Trolox were employed as standards. The results were expressed in mmol of Trolox equivalent per gram of dry weight (mmol TE/g dw).

### 3.6. Anti-Aging Activity

The inhibition of elastase and hyaluronidase enzymes was measured to evaluate anti-aging activity. For this analysis, the extract was obtained using a rotative evaporator (Bibby Scientific Limited, Stone, Staffordshire, UK) and then freeze-dried in BenchTop Pro with omnitronics, SP Scientific, Warminster, PA, USA)). Following Leal et al. [18], 1 mg of hydro methanolic extract (70:30, *v/v*) was dissolved in 1 mL of 10% DMSO.

#### 3.6.1. Elastase Inhibition 

To determine elastase inhibition, 50 µL of grape stem extracts (dissolved in 10% DMSO at 1 mg/mL) or negative control (Tris-HCl buffer 0.2 mM, pH 8) was added to 160 µL of buffer in 96-well plates. Then, 20 µL of N-Succinyl-Ala-Ala-Ala-*p*-nitroanilide (substrate) was added to the mixture. Afterwards, the mixture was incubated at room temperature, protected from light, for 10 min. Finally, 20 µL of elastase (1 U/mL) was added and the absorbance was measured at 410 nm using a microplate reader. This assay was calculated according to the following equation:

Elastase Inhibition (%) = [(Abs_410_ control − Abs_410_ sample)/(Abs_410_ control)] × 100 (5)

#### 3.6.2. Hyaluronidase Inhibition

For the hyaluronidase inhibition assay, 140 µL of Tris-HCl buffer (50 mM, pH 7.0) was initially mixed with 20 µL of hyaluronidase (4 mg/mL), 20 µL of hyaluronic acid sodium salt (0.4 mg/mL), and 20 µL of the grape stem extracts or 20 µL of Tris-HCl buffer as the negative control. The mixture was then incubated at 37 °C for 60 min. After incubation, the microplate was left to cool to room temperature. Finally, 20 µL of cetylpyridinium chloride solution (10%) was added and the absorbance was measured at 600 nm using a microplate reader. This enzymatic inhibition (%) was calculated according to the following equation:

Hyaluronidase Inhibition (%) = [(Abs_600_ control − Abs_600_ sample)/(Abs_600_ control)] × 100 (6)

### 3.7. Skin Depigmenting Activity

#### Tyrosinase Inhibition

To assess tyrosinase inhibition, 10 µL of grape stem extract, 10 µL of 10% DMSO (negative control), or 10 µL of Kojic acid (positive control) was added to a microplate. Subsequently, 20 µL of tyrosinase (1000 U/mL) and 170 µL of a mixture containing L-tyrosine solution, phosphate buffer (50 mM, pH 6.5), and distilled water in a 10:10:9 ratio was added. The mixtures were incubated at 37 °C for 10 min, after which the absorbance was measured at 490 nm using a microplate reader. This assay was calculated according to the following equation:

Tyrosinase Inhibition (%) = [(Abs_490_ control − Abs_490_ sample)/(Abs_490_ control)] × 100 (7)

### 3.8. Polyphenolic Profile and Quantification of Grape Stems

The quantitative polyphenolic profile of the grape stems from the seven white varieties was achieved by applying the methodology described by Costa-Pérez et al. [17]. 

The chromatographic separation of polyphenols was developed on a Luna C18 column (250.0 × 4.6 mm, 5.0 μm particle size, Phenomenex, Macclesfield, UK) using an Agilent HPLC 1100 series (Agilent Technologies, Waldbronn, Germany) that consisted of a binary pump (model G1312A), an autosampler (model G1313A), a degasser (model G1322A), a photodiode array (PDA) detector (model G1315B), and an ion trap spectrometer (model G2445A) with an electrospray ionization interface, all controlled by LCMSD software, v. 4.1 (Agilent Technologies), operated according the specifications detailed by Barros et al. [31]. The mobile phases used were dH_2_O/formic acid (99.0:1.0, *v/v*) (solvent A) and acetonitrile/formic acid (9.0:1.0, *v/v*) (solvent B). Spectral data from all peaks were detected in the 200–600 nm range, with chromatograms recorded at 280 nm for proanthocyanidins, 330 nm for phenolic acids and stilbenes, and 360 nm for flavonols. Mass spectrometry data were acquired in the negative mode. Phenolic compound identification was carried out by examining the retention time (min), parent ions, and MS2 fragmentation patterns. Phenolic compounds were quantified using PDA chromatograms recorded at 280 nm for proanthocyanidins, 330 nm for phenolic acids, and 360 nm for flavonols, applying daily prepared calibration curves with catechin (proanthocyanidins and catechin derivatives), chlorogenic acid (phenolic acids), and quercetin glucoside (flavonols), and the concentration expressed as µg/g dw.

### 3.9. Statistical Analysis

All results are all provided as the mean ± standard deviation (SD) for triplicate (*n* = 3) analyses. For the analysis of phenolic content, antioxidant capacity, and tyrosinase inhibition, the non-parametric Kruskal–Wallis test was employed due to the lack of homogeneity of variances and data normality. The Conover–Iman post hoc test was used for the first two analyses, while the Dunn post hoc test was applied for tyrosinase inhibition. For analysing differences in polyphenolic quantification and elastase inhibition, where the data were normally distributed but variances were not homogeneous, the Welch test was employed. For polyphenolic quantification, the Games–Howell post hoc test was used, while for elastase inhibition, the Dunn post hoc test was applied. These analyses were performed using GraphPad Prism 10 (GraphPad Software, San Diego, CA, USA). Principal Component Analysis (PCA) was conducted using the mean values from triplicate measurements in JMP Statistical Discovery TM, version 11.0.0 (Neil Hodgson, Cary, NC, USA). The data were normalized to a range of 0–100, considering the highest mean value observed in each experiment. The heatmap of correlations between the phenolic composition, antioxidant capacity, anti-aging, and skin depigmentation activities was generated using GraphPad Prism, version 10 (GraphPad Software, San Diego, CA, USA).

## 4. Conclusions

This study highlights the potential of white grape (*Vitis vinifera* L.) stems as valuable by-products for cosmetic and pharmaceutical applications, attributed to their polyphenolic composition. Notably, the results revealed considerable concentrations of proanthocyanidins and catechin derivatives, phenolic acids, and flavonols, being the first study exploration of the varieties ‘Síria’, ‘Gouveio Real’, and ‘Códega do Larinho’. The last one exhibited the highest levels of polyphenolic compounds, including proanthocyanidin dimer digallate (B-type), catechin-gallocatechin, proanthocyanidin dimer (B-type) Isomers 2 and 3, and *trans*-caftaric acid. This variety also demonstrated some of the highest results in reducing power, the highest results in radical scavenging capacity, and elastase and tyrosinase inhibition. Our findings reveal a significant positive correlation between some proanthocyanidins and catechin derivatives and the DPPH and FRAP methodologies. A positive correlation was also demonstrated between flavonols and elastase inhibition, highlighting the health-promoting potential of grape stem extracts. These results promote the valorisation of grape stems, suggesting their use in developing innovative products with potential health benefits. Future research should aim to further elucidate the bioactive properties and potential applications of these phenolic compounds across various industries.

## Figures and Tables

**Table 1:** Total phenolic, *ortho*-diphenol, and flavonoid contents.

Varieties	Total Phenolics (mg GAE/g dw)	*Ortho*-Diphenols (mg GAE/g dw)	Flavonoids (mg CE/g dw)
‘Códega do Larinho’	49.98 ± 0.94 a	40.75 ± 2.56 a	32.15 ± 1.30 b
‘Viosinho’	31.70 ± 0.87 d	26.67 ± 0.59 c	17.54 ± 0.66 g
‘Malvasia Fina’	34.11 ± 0.90 cd	30.62 ± 0.42 b	22.32 ± 0.69 e
‘Síria’	45.27 ± 3.90 ab	40.23 ± 3.49 a	35.91 ± 1.75 a
‘Gouveio Real’	38.97 ± 0.21 b	40.85 ± 0.13 a	29.99 ± 1.30 c
‘Arinto’	38.13 ± 0.24 c	31.37 ± 1.67 b	26.93 ± 1.79 d
‘Moscatel’	26.09 ± 0.60 e	25.92 ± 0.67 c	20.77 ± 0.17 f

GAE, gallic acid equivalents; CE, catechin equivalents; dw, dry weight. Data are presented as mean ± SD (*n* = 3). Distinct letters in the same column correspond to significant differences between varieties (*p* < 0.05) according to a Kruskal–Wallis test followed by a post hoc Conover–Iman test.

**Table 2:** Identification of polyphenols grape (*Vitis vinifera* L.) stems of ‘Códega do Larinho’, ‘Viosinho’, ‘Malvasia Fina’, ‘Síria’, ‘Gouveio Real’, ‘Arinto’, and ‘Moscatel’, by HPLC-PDA-ESI-MSn operated in negative mode.

Compounds	*m/z* [M − H]^−^	*m/z* MS2 [M − H]^−^
Proanthocyanidins and Catechin Derivatives		
Proanthocyanidin dimer digallate (B-type)	881	695 (100), 289 (16), 443 (16)
Proanthocyanidin trimer (B-type) (Isomer 1)	865	695 (100), 405 (36), 287 (29), 577 (27)
Catechin–gallocatechin	593	441 (100), 423 (15), 407 (12)
Proanthocyanidin dimer (B-type) (Isomer 1)	577	425 (100), 407 (97), 289 (29), 451 (16)
Proanthocyanidin dimer (B-type) (Isomer 2)	577	425 (100), 407 (97), 289 (29), 451 (16)
Proanthocyanidin dimer (B-type) (Isomer 3)	577	425 (100), 407 (97), 289 (29), 451 (16)
Proanthocyanidin trimer (B-type) (Isomer 2)	865	695 (100), 405 (36), 287 (29), 577 (27)
Catechin	289	245 (100), 205 (31)
Proanthocyanidin dimer (B-type) (Isomer 4)	577	425 (100), 407 (97), 289 (29), 451 (16)
**Phenolic acids**		
*trans*-caftaric acid	311	149 (100), 179 (38), 135 (8)
*trans*-coutaric acid	295	163 (100)
**Flavonols**		
Quercetin-3-*O*-glucuronide	477	301 (100), 151 (2)
Kaempferol-3-*O*-glucoside	447	285 (100), 303 (53), 151 (15), 179 (6)

**Table 3:** Quantification by HPLC-DAD-ESI-MS/Mn of polyphenolic compounds present in white grape (*Vitis vinifera* L.) stems.

Rt (min)	λ (nm)	Quantified Compounds	White Grape (*Vitis vinifera* L.) Stems Varieties						
			‘Códega do Larinho’	‘Viosinho’	‘Malvasia Fina’	‘Síria’	‘Gouveio Real’	‘Arinto’	‘Moscatel’
**Proanthocyanidins and catechin derivatives (mg/g dw)**									
5.8	280	Proanthocyanidin dimer digallate (B-type)	255.60 ± 11.14 a	157.47 ± 4.27 c	171.34 ± 19.55 bc	158.57 ± 24.2 bc	177.48 ± 22.04 bc	177.59 ± 5.87 b	168.39 ± 11.46 bc
6.7	280	Proanthocyanidin trimer (B-type) (isomer 1)	225.57 ± 7.52 c	574.27 ± 52.76 a	283.22 ± 38.86 bc	218.79 ± 10.09 c	322.03 ± 13.02 b	291.64 ± 14.79 b	510.00 ± 29.41 a
7.4	280	Catechin-gallocatechin	206.84 ± 16.52 ab	131.14 ± 9.08 c	180.54 ± 22.67 abc	221.39 ± 12.3 a	179.46 ± 4.69 bc	201.14 ± 4.11 ab	176.74 ± 8.76 bc
9.7	280	Proanthocyanidin dimer (B-type) (isomer 1)	<LOQ	<LOQ	<LOQ	<LOQ	<LOQ	<LOQ	<LOQ
10.0	280	Proanthocyanidin dimer (B-type) (isomer 2)	670.35 ± 75.01 a	342.04 ± 41.94 b	495.38 ± 49.09 a	903.37 ± 153.29 a	626.40 ± 41.40 a	838.77 ± 139.16 a	882.14 ± 50.55 a
10.8	280	Proanthocyanidin dimer (B-type) (isomer 3)	714.59 ± 103.88 a	191.52 ± 18.16 d	253.25 ± 38.56 bcd	327.57 ± 18.34 b	263.56 ± 10.57 c	473.15 ± 35.58 a	680.42 ± 44.77 a
11.0	280	Proanthocyanidin trimer (B-type) (isomer 2)	168.16 ± 5.47 a	144.38 ± 32.24 a	156.59 ± 5.64 a	197.88 ± 7.68 a	169.38 ± 10.63 a	235.73 ± 13.75 a	272.23 ± 64.96 a
11.4	280	Catechin	430.26 ± 33.16 c	325.67 ± 16.01 d	504.74 ± 32.98 bc	645.50 ± 23.76 a	691.86 ± 70.05 a	543.32 ± 31.50 ab	274.42 ± 25.87 d
12.3	280	Proanthocyanidin dimer (B-type) (isomer 4)	179.53 ± 6.91 a	156.40 ± 18.89 a	169.83 ± 18.60 a	192.93 ± 11.30 a	191.12 ± 21.97 a	173.34 ± 5.62 a	198.68 ± 14.32 a
		**Total**	2850.91 ± 253.76 ab	2022.90 ± 170.48 c	2214.90 ± 154.36 bc	2866.00 ± 229.40 a	2621.28 ± 175.16 ab	2934.67 ± 232.59 a	3163.02 ± 221.14 a
**Phenolic Acids (µg/g dw)**									
10.5	330	*trans*-caftaric acid	174.69 ± 21.15 ab	65.44 ± 32.93 cde	50.19 ± 6.26 e	66.07 ± 8.45 de	76.56 ± 4.44 d	124.49 ± 7.45 bc	226.22 ± 16.75 a
12.7	330	*trans*-Coutaric acid	52.93 ± 8.35 c	131.58 ± 5.14 a	25.63 ± 1.56 d	22.95 ± 0.96 de	14.01 ± 3.95 e	21.20 ± 1.33 e	83.97 ± 6.57 b
		**Total**	227.62 ± 29.45 b	197.02 ± 34.23 b	75.82 ± 6.70 c	89.02 ± 8.44 c	90.57 ± 6.49 c	145.69 ± 8.55 b	310.19 ± 18.95 a
**Flavonols (µg/g dw)**									
16.8	360	Quercetin-3-*O*-glucuronide	21.16 ± 1.32 b	ND	39.08 ± 4.17 a	39.93 ± 4.67 a	34.02 ± 4.44 ab	35.90 ± 6.30 ab	30.05 ± 2.03 ab
17.4	360	Kaempferol-3-*O*-glucoside	216.21 ± 15.10 d	327.76 ± 23.03 c	537.50 ± 28.52 a	380.10 ± 46.13 bc	353.52 ± 24.85 bc	371.89 ± 36.83 bc	448.45 ± 39.06 ab
		**Total**	237.37 ± 15.88 d	327.76 ± 23.03 c	576.58 ± 31.67 a	420.03 ± 43.85 bc	387.54 ± 24.62 bc	407.79 ± 42.90 bc	478.50 ± 39.49 ab

ND, not detected; Rt, retention time; dw, dry weight, LOQ, limit of quantification. Distinct letters in the same row indicate significant differences between varieties (*p* < 0.05), according to the Welch test followed by a post hoc Games–Howell test.

**Table 4:** Radical scavenging capacity and reducing power of the polyphenolic burden of the grape stems.

Variety	DPPH Assay (mmol TE/g dw)	ABTS Assay (mmol TE/g dw)	FRAP Assay (mmol TE/g dw)
‘Códega do Larinho’	0.32 ± 0.02 a	0.46 ± 0.03 a	0.34 ± 0.01 ab
‘Viosinho’	0.12 ± 0.01 e	0.37 ± 0.03 b	0.21 ± 0.00 f
‘Malvasia Fina’	0.22 ± 0.00 d	0.20 ± 0.02 cd	0.25 ± 0.01 d
‘Síria’	0.26 ± 0.01 bc	0.46 ± 0.02 a	0.36 ± 0.01 a
‘Gouveio Real’	0.25 ± 0.01 bc	0.20 ± 0.01 cd	0.33 ± 0.01 b
‘Arinto’	0.24 ± 0.00 c	0.18 ± 0.02 d	0.29 ± 0.02 c
‘Moscatel’	0.22 ± 0.01 d	0.23 ± 0.00 c	0.22 ± 0.00 e

DPPH, DPPH^●^ scavenging capacity; ABTS, ABTS^●+^ scavenging capacity; FRAP, ferric-reducing antioxidant power; TE, Trolox equivalents. Data are presented as mean ± SD (*n* = 3). Distinct letters within the same column correspond to significant differences between varieties (*p* < 0.05) according to a Kruskal–Wallis test followed by a post hoc Conover–Iman test.

**Table 5:** Sampling of white grape (*Vitis vinifera* L.) stems from the *Região Demarcada do Douro*.

Variety	Sample Code	Location	Coordinates
‘Códega do Larinho’	CL	Santa Marta Penaguião	41°12′39″ N 7°47′04″ O
‘Viosinho’	V	Santa Marta Penaguião	41°12′39″ N 7°47′04″ O
‘Malvasia Fina’	MF	Penajóia	41°8′24″ N 7°51′12″ O
‘Síria’	S	Santa Marta Penaguião	41°12′39″ N 7°47′04″ O
‘Gouveio Real’	GR	Medrões	41°12′35″ N 7°49′18″ O
‘Arinto’	A	Medrões	41°12′35″ N 7°49′18″ O
‘Moscatel’	M	Medrões	41°12′35″ N 7°49′18″ O

## Data Availability

Data are contained within the article.
